# Baicalin regulates the dopamine system to control the core symptoms of ADHD

**DOI:** 10.1186/s13041-019-0428-5

**Published:** 2019-02-08

**Authors:** Rongyi Zhou, Jiaojiao Wang, Xinmin Han, Bingxiang Ma, Haixia Yuan, Yuchen Song

**Affiliations:** 1 The First Affiliated Hospital of Henan University of Chinese Medicine, Renmin road no.19, Jinshui District, Zhengzhou City, 450000 Henan Province China; 2 Nanjing University of Chinese Medicine, Xianlin road no.138, Qixia District, Nanjing City, Jiangsu Province, 210023 China

**Keywords:** Baicalin, ADHD, Dopamine, Spontaneously hypertensive rats, Behavioral tests

## Abstract

We aimed to test the therapeutic effects of baicalin on attention deficit hyperactivity disorder (ADHD) in an animal model and to explain the potential mechanism. We investigated the therapeutic effects and mechanisms of baicalin in a spontaneously hypertensive rat (SHR) model of ADHD depending on the dopamine (DA) deficit theory. In this study, fifty SHRs were randomly divided into five groups: methylphenidate (MPH), baicalin (50 mg/kg, 100 mg/kg, or 150 mg/kg), and saline-treated. Ten Wistar Kyoto (WKY) rats were used as controls. All rats were orally administered the treatment for four weeks. Motor activity, spatial learning and memory ability were assessed with the open-field and Morris water-maze tests. The mRNA and protein levels of tyrosine hydroxylase (TH), vesicular monoamine transporter 2 (VMAT2), synaptosomal-associated protein of molecular mass 25kD (SNAP25) and synataxin 1a in synaptosomes were detected with real-time polymerase chain reaction (PCR) and Western blot. In addition, DA levels were measured in the prefrontal cortex and striatum. The results indicated that both MPH and baicalin at doses of 150 mg/kg and 100 mg/kg significantly decreased the hyperactivity and improved the spatial learning memory deficit in the SHRs and increased the synaptosomal mRNA and protein levels of TH, SNAP25, VMAT2 and synataxin 1a compared with saline treatment. MPH significantly increased DA levels in both the prefrontal cortex (PFC) and striatum, while baicalin significantly increased DA levels only in the striatum. The results of the present study showed that baicalin treatment was effective for controlling the core symptoms of ADHD. Baicalin increased DA levels only in the striatum, which suggested that baicalin may target the striatum. The increased DA levels may partially be attributed to the increased mRNA and protein expression of TH, SNAP25, VMAT2, and syntaxin 1a. Therefore, these results suggested that the pharmacological effects of baicalin were associated with the synthesis, vesicular localization, and release of DA and might be effective in treating ADHD. However, further studies are required to better understand the molecular mechanisms underlying these findings.

## Background

ADHD, which has the core symptoms of inattention, hyperactivity, and/or increased impulsivity [1], is a common childhood neurodevelopment disorder with a 5.9% global annual incidence [2]. In addition, approximately 25% of adults who were diagnosed with ADHD in childhood continue to show symptoms. Studies have indicated that adults with ADHD have a significant risk of suicide and negative labor market outcomes [3, 4]. Therefore, treating children with ADHD is important for society. However, the underlying pathogenesis of ADHD remains unclear. It is widely accepted that the dysfunction of catecholamine and particular DA neuronal systems (the dopaminergic hypothesis) contributes to the pathophysiology of ADHD [5]. Currently, psychotropic drugs such as MPH, a DA transporter (DAT) inhibitor, are the first choice for the clinical treatment of ADHD. Fortunately, many studies have reported that the drug is effective in controlling the core symptoms in ADHD [3]; however, this drug has been reported to have many adverse reactions, such as loss of appetite, induced tics, drug dependence, and increased heart rate, that easily rebound after stopping treatment [6], which brings certain limitations to clinical medication. This situation makes it difficult for doctors and parents to make choices. Therefore, exploring and providing new options for clinical treatment of ADHD is necessary.

Baicalin is a flavonoid purified from the plant *Scutellaria baicalensis* Georgi [7]*.* Baicalin is the main medicinal component of the plant, the highest concentration is found in the *Radix scutellariae* [8]. *Scutellaria baicalensis* has been used for thousands of years in traditional Chinese medicine to treat inflammation [9], fever, jaundice [10], and hypertension. In recent studies, baicalin showed effectively protective effects on 6-hydroxydopamine (6-OHDA), 1-methyl-4-phenylpyridinium (MPP+), 1-mehtyl-4-phenyl-1,2,3,6-tetrahydropyridine (MPTP) and methamphetamine-induced neurotoxicity in cell lines and animal models [11–14] via anti-inflammatory, neuroprotective, and antioxidant effects [15–17] that were closely related to central nervous system (CNS) diseases. Moreover, researchers have found that baicalin can rapidly pass through the blood-brain barrier (BBB) [18] and shows neuroprotective and synaptoprotective effects on DA neurons [19] and can be used to treat DA dysfunction-associated neurodegenerative diseases, such as Alzheimer’s disease (AD) and Parkinson’s disease [20] via its neuroprotective and cognitive enhancement effects [11, 21]. Furthermore, Chen et al. reported that baicalin targets the DA system [22]. We hypothesized that baicalin regulates the DA system in the brain and would be therapeutic for ADHD [23]. In this study, we performed a series of experiments to test the hypothesis of the therapeutic effects of baicalin on ADHD animal models and explained the potential mechanisms dependent upon the dopamine deficit theory.

## Materials and methods

### Animals

Three- to four-week-old male SHRs (*n* = 50) and WKY rats (*n* = 10) (both from Charles River Laboratories) (Beijing Vital River Laboratory Animal Technology Co., Ltd., Beijing, China) were used in this study [experimental animal production license SCXK(J)2012–0001]. All animals were housed and maintained on a 12 h on/12 h off light/dark cycle in a specific pathogen-free (SPF) laboratory in the Animal Center of the Nanjing University of Chinese Medicine [SYSK(SU)2014–0001]. The temperature and humidity were maintained at 20–24 °C and 45–55%, respectively. All animals were allowed free access to food and water, and all efforts were made to minimize animal suffering and to reduce the numbers of animals used. All procedures were approved by the Institutional Animal Care and Use Committee of the Nanjing University of Chinese Medicine.

### Groups and drugs

The WKY rats served as the control group (*n* = 10). The SHR rats were randomly divided into the following five groups with 10 rats per group: model group (treated with saline), methylphenidate (MPH, 1.5 mg/kg) group [24], baicalin (50 mg/kg) group, baicalin (100 mg/kg) group, and baicalin (150 mg/kg) group. These doses of baicalin were based on previous studies that showed that baicalin doses of approximately 100 mg/kg significantly affected DA in the brain [21, 22]. The MPH was obtained from Xi’an Janssen Pharmaceutical Co., Ltd. (Xi’an, China; Import permit number TP120120504). Baicalin was purchased from Chengdu Pufei De Biotech Co., Ltd. (Chengdu, China; product number JOT-10027), and its purity was 95.46% (as detected by high-performance liquid chromatography [HPLC] at 280 nm). MPH and baicalin were administered orally. The control (WKY rats) and model groups were administered equal amounts of saline (1.5 mL/100 g) twice a day (08:00–09:00 am and 13:30–14:30 pm) for four weeks. The weight of the rats and the amount of remaining feed were weighed before daily gavage, and the amount of gavage was calculated according to the body weight. All drugs were warmed to room temperature before administration. All gavage appliances were covered with white medical tape to avoid any effects of the different colors of the drugs.

### Open-field test

The open-field arena consisted of a black iron box (100 × 100 × 40 cm) divided into 16 squares of 25 × 25 cm (12 peripheral squares and four central squares). A 60-watt light was located one meter above the floor of the arena. Animals were gently and individually placed into the center of the open-field box. The moving distance and movement velocity were recorded by the small animal activities record analysis system (SLY-ETS version 1.66; Beijing Sunny Instruments Co. Ltd., Beijing, China) for five min per test. All rats were tested once per week. After each test, the open-field arena was cleaned with a disinfectant, then with 70% ethanol and water, and then blow-dried.

### Morris water maze (MWM) test

The MWM (Beijing Sunny Instruments Co. Ltd., Beijing, China) is composed of a monitor with the video camera set in the ceiling, a computerized tracking system, water temperature automatic control system, and a black circular metal pool (150 cm in diameter, 60 cm in height) filled with water (24 ± 2 °C) and pasted with distinctive visual cues (different geometric patterns pasted on the pool). A black metal platform with a roughened surface (12 cm in diameter and 50 cm in height) was located in the center of a fixed quadrant in southeast and submerged to approximately 0.7 to 1 cm below the water surface (the platform was placed southeast during the first five days of training and removed on the sixth day). Rats were placed in the pool for 120 s and allowed to swim freely to become familiar with the training environment before the experiment. During the first five days, the rats received training of 4 trials per day for 5 consecutive days. The intervals between trials were 60 s, each session lasted for 2 min, after which the rat was removed from the pool. In each trial, the rats had to swim until they climbed onto the platform submerged underneath the water. On the sixth day, the platform was removed (probe trial) from the pool. Rats were placed in the northwest quadrant and allowed to swim twice for 120 s. The duration from the time when the rats entered the water to the time when they climbed onto the platform was recorded and defined as the escape latency. Swimming distance and average speed were also recorded. In the probe trial, number of platform crosses (times), the ratio of target quadrant swimming time and distance to the total swimming time and distance were recorded by a small animal activity record analysis system (Beijing Sunny Instruments Co. Ltd., SLY-ETS version 1.66; Beijing, China) to evaluate learning and memory [25]. At the end of each training session, each rat was dried with a towel and an electric heater before it was returned to its home cage.

### Synaptosome preparation

Since DA synthesis and vesicular localization and release were all carried out in the synaptosomes, we prepared synaptosomes as the experimental specimens. Synaptosomes were prepared using a rapid Percoll gradient procedure previously reported by Peter R Dunkley [26]. Gradient solutions were prepared with 3, 10, 15, and 23% (*v*/v) Percoll® (Sigma-Aldrich Co. LLC, St. Louis, MO, USA). Brain tissue was homogenized for 20–30 s using a ball mill instrument (MM 400; Retsch GmbH, Haan, Germany) at a rate of 20 times/s and centrifuged at 3600 g for 10 min at 4 °C (5427 R; Eppendorf AG, Wesseling, Germany). The supernatant fraction was collected and applied to a discontinuous Percoll® gradient and centrifuged for 5 min (Allegra 64R; Beckman Coulter, Inc., Brea, CA, USA) at 12000 g. The fractions were collected from the interfaces between the Percoll® layers at the 10 to 23% gradient level, diluted, centrifuged to remove Percoll®, and subsequently resuspended in a physiological buffer for observational and functional analyses. The morphological structure of the synaptosomes was identified with electron microscope (S-300 N; Hitachi High-Technologies Corporation, Tokyo, Japan) to confirm the experimental specimens were synaptosomes.

### Western blotting

Synaptosomes were homogenized in ice-cold RIPA lysis buffer for 30 min. The protein concentration in the lysates was evaluated using a BCA protein assay kit (Thermo Fisher Scientific Inc., Waltham, MA, USA). Protein samples were separated on an SDS-PAGE gel by subjecting it to electrophoresis, and proteins were subsequently transferred to presoaked polyvinylidene difluoride (PVDF) membranes (Bio-Rad Laboratories, Inc., Hercules, CA, USA). PVDF membranes were blocked in 5% milk in PBS containing 0.1% Tween 20, at room temperature and subsequently incubated with the following primary antibodies: anti-Tyrosine Hydroxylase (TH) antibody (ab6211, 1:200; Abcam plc, Cambridge, UK), anti-SNAP25 antibody (ab5666, 1:100; Abcam plc), anti-Syntaxin 1a antibody (ab41453, 1:100; Abcam plc), anti-VMAT2 antibody (ab70808, 1:500; Abcam plc), and anti-β-actin antibody (sc-47,778, 1:1000; Santa Cruz Biotechnology, Inc., Dallas, TX, USA) overnight at 4 °C. The blots were washed and incubated with horseradish peroxidase-conjugated secondary antibody at room temperature. Band detection was performed using the enhanced chemiluminescence (ECL) detection kit (Thermo Fisher Scientific, MA, USA). The intensity of the detected bands was calculated densitometrically using Image-ProR Plus 5.0 software (Media Cybernetics, Bethesda, MD, USA). Data were normalized to β-actin levels to avoid any effects of possible variations in protein expression.

### Real-time polymerase chain reaction (PCR)

The total RNA was extracted from the synaptosomes with TRIzol reagent (Takara Bio, Inc., Dalian, China) according to the manufacturer’s instructions. The RNA was reverse transcribed to cDNA by using a Takara PrimeScript™ 1st Stand cDNA Synthesis Kit (Takara Bio, Inc.), and the obtained cDNA was used as a template to perform PCR amplification with a SYBR® Premix Ex TaqTM II kit (Takara Bio, Inc.). The specific primers were as follows: TH, sense 5′-CGAGCTGTGAAGGTGTTTGA-3′ and antisense, 5′-GTACACCTGGTCCGAGAAGC-3′; SNAP25, sense 5′-ATCTGGCGATTCTGGGTGT-3′ and antisense 5′-CGGGAAAATGAAATGGATGA-3′; VMAT2, sense 5′-CTGGTCTGGTGGTCTGGATT-3′ and antisense 5′-CTGTTCATCGTGTTCCTTGC-3′; syntaxin 1a, sense 5′-GACGAGCGGTTCAGACCTT-3′ and antisense 5′-ACCCCGATGAGAAGACCAA-3′; and glyceraldehyde 3-phosphate dehydrogenase, sense, 5-GACATGCCGCCTGGAGAAAC-3 and antisense, 5-AGCCCAGGATGCCCTTTAGT-3. Each 20-μL reaction consisted of 2 μL of cDNA, 10 μL of SYBR® Premix Ex Taq II, and 10 μmol/L of both the sense and antisense primers. Three replicates of each PCR run were performed. The mRNA levels of all target genes in each sample were normalized to the levels of glyceraldehyde 3-phosphate dehydrogenase by using the Delta-delta Ct method.

### DA content in the PFC and striatum [25]

Rat PFC and striatum tissues (approximately 20 mg) were weighed and sonicated in 100 μL of a 0.1 M HClO_4_/10 μM ascorbate solution for 1 min. Brain samples were centrifuged at 12000 *g* for 10 min at 4 °C. To determine DA concentrations, samples were eluted with a mobile phase containing 25 mM acetate buffer with 0.75 mM sodium heptanesulfonate (pH 3.9)-methanol (85:15, *v*/v) on a Hypersil ODS column (250 × 4.6 mm, 5 μm) with the flow rate set at 1.0 mL/min. A 20-μL aliquot was injected into a HPLC system equipped with a fluorescence detector (Waters, USA). The excitation and emission wavelengths were set at 305 nm and 360 nm, respectively.

### Statistical analysis

All data were analyzed with SPSS software (version 19.0; IBM Corporation, Armonk, NY, USA). The data are expressed as the mean ± standard error of the mean. The differences among the groups were evaluated statistically with one-way analyses of variance and post hoc Fischer’s LSD tests and least significant difference posthoc tests when appropriate. Differences with *p* values less than 0.05 were considered statistically significant.

## Results

### Effect of baicalin on the growth and development of rats

All data for the different groups are shown in Tables 1 and 2. One-way ANOVA showed between-group differences in weight (*F*_(5,55)_ = 10.278, *p* = 0.000) and food intake (*F*_(5,55)_ = 15.781, *p* = 0.002). Before the start of the experiment (week 0), there was no difference in weight and food intake between the groups. In the first week of the experiment, compared with the WKY + saline group, the weight of the MPH + saline group increased significantly (*p =* 0.036), and the food intake of the SHR + baicalin (150 mg/kg) group was significantly higher than that of the WKY + saline group and the MPH + saline group (*p =* 0.015, LSD). In the second week of the experiment, compared with the WKY + saline group, the weight of the other groups was significantly increased (*p =* 0.004, LSD), and the food intake of the SHR + baicalin(150 mg/kg) group was significantly higher than the other groups (*p =* 0.023, LSD). In the third week of the experiment, compared with the WKY + saline group, except for the SHR + baicalin(50 mg/kg) group, the body weight of the other groups was significantly increased (*p =* 0.006, LSD), and the food intake in the SHR + baicalin(150 mg/kg) group was significantly higher than the other groups (*p <* 0.001, LSD); In the 4th week of the experiment, compared with the WKY + saline group, the weight of the other groups increased significantly (*p <* 0.001, LSD). Compared with the MPH + saline group, the weight in the SHR + baicalin(150 mg/kg) group increased significantly (*p <* 0.001, LSD); the food intake in the SHR + baicalin(150 mg/kg) group was significantly higher than the other groups (*p <* 0.001, LSD), as shown in Tables 1 and 2.

**Table 1:** Changes in rats weight (mean ± SEM, g, *n* = 10)

Group	Week 0	Week 1	Week 2	Week 3	Week 4
WKY + saline	70.26 ± 2.03	92.96 ± 7.15	114.64 ± 7.27	136.83 ± 7.73	169.95 ± 12.45
SHR + saline	72.31 ± 3.48	99.11 ± 10.26	122.40 ± 12.32^#^	149.08 ± 17.50^#^	176.41 ± 13.05^#^
SHR + MPH(1.5 mg/kg)	72.01 ± 2.85	105.47 ± 8.09 ^#^	128.18 ± 7.98^#*^	153.15 ± 8.76^#^	180.54 ± 11.57^#^
SHR + baicalin(50 mg/kg)	71.35 ± 1.19	98.91 ± 8.07	119.78 ± 8.75^#^	142.31 ± 11.13	176.05 ± 12.47^#^
SHR + baicalin(100 mg/kg)	72.72 ± 3.24	100.80 ± 6.71	122.33 ± 8.13^#^	144.49 ± 10.97^#^	177.21 ± 10.77^#^
SHR + baicalin(150 mg/kg)	74.03 ± 6.60	102.96 ± 7.72	125.59 ± 9.78^#^	152.50 ± 12.9^#^	183.95 ± 14.07^#*^

Note: Comparison of the changes in weight in all groups; values are expressed as the mean ± S.E.M. ^#^ indicated changes in rats weight in other groups compared with WKY+saline group; ^*^ indicated changes in rats weight in other groups compared with SHR+saline group

**Table 2:** Changes in food intake (mean ± SEM, g, *n* = 10)

Group	Week 0	Week 1	Week 2	Week 3	Week 4
WKY + saline	8.06 ± 1.0	10.08 ± 1.41	12.11 ± 1.91	14.10 ± 4.47	15.97 ± 1.83
SHR + saline	9.11 ± 0.97	11.70 ± 1.62	12.96 ± 1.89	15.75 ± 3.73	17.37 ± 3.12
SHR + MPH(1.5 mg/kg)	9.14 ± 1.03	12.37 ± 2.26	13.60 ± 1.84	15.89 ± 3.55	17.83 ± 3.59
SHR + baicalin(50 mg/kg)	9.01 ± 0.78	11.05 ± 2.68	12.33 ± 2.05	14.02 ± 2.99	17.19 ± 4.63
SHR + baicalin(100 mg/kg)	8.97 ± 1.12	11.99 ± 2.63	13.28 ± 2.03	14.51 ± 3.39	17.07 ± 2.36
SHR + baicalin(150 mg/kg)	9.34 ± 1.10	16.44 ± 1.3^#*^	19.83 ± 2.06^#*^	22.63 ± 3.42^#*^	25.71 ± 4.60^#*^

Note: Comparison of the changes in food intake in all groups; values are expressed as the mean ± S.E.M. ^#^ indicated changes in food intake in other groups compared with WKY+saline group; ^*^ indicated changes in food intake in other groups compared with SHR+saline group

#### Baicalin treatment-induced decrease in SHR motor activity

All datas for the different groups are shown in Tables 3 and 4. One-way ANOVA showed between-group differences in moving distance (*F*_(5,55)_ = 13.639, *p <* 0.001) and moving speed (*F*_(5,55)_ = 24.605, *p* = 0.000). As shown in Table 3 and Table 4, the moving distance (*p <* 0.01, LSD) and moving speed (*p <* 0.01, LSD) were significantly increased in the SHRs compared with the WKY + saline group before drug treatment. After one week of treatment, the moving distance and moving speed in MPH + saline (*p <* 0.01, LSD) and SHR + baicalin (150 mg/kg) group (*p* < 0.05, LSD) were significantly decreased compared to SHR + saline treated. No significant differences were observed in the other groups compared to the SHR + saline group.

**Table 3:** Moving distance in the open field test (mean ± SEM, m, n = 10)

Group	Week 0	Week 1	Week 2	Week 3	Week 4
WKY + saline	21.66 ± 2.67	19.47 ± 2.24	15.16 ± 2.27	12.55 ± 1.63	9.78 ± 2.75
SHR + saline	43.84 ± 2.39^##^	42.18 ± 2.03^##^	40.04 ± 2.35^##^	39.60 ± 2.22^##^	40.51 ± 1.79^##^
SHR + MPH(1.5 mg/kg)	46.34 ± 3.13	26.66 ± 2.67^**^	21.14 ± 2.30^**^	17.69 ± 2.46^**^	12.23 ± 2.22^**^
SHR + baicalin(50 mg/kg)	45.06 ± 1.48	41.57 ± 1.75	38.89 ± 2.71	29.12 ± 2.57^*^	23.41 ± 2.87^**^
SHR + baicalin(100 mg/kg)	44.89 ± 3.42	41.01 ± 2.82	31.64 ± 2.58^*^	26.77 ± 1.90^**^	21.19 ± 3.48^**^
SHR + baicalin(150 mg/kg)	46.48 ± 2.80	33.24 ± 1.75^*^	24.54 ± 1.63^**^	17.52 ± 1.76^**^	14.26 ± 2.83^**^

Note: Comparison of the moving distance in the open field test in all groups; values are expressed as the mean ± S.E.M. ^#^*p* < 0.05 and ^##^*p* < 0.01 versus WKY + saline group; ^*^p < 0.05 and ^**^p < 0.01 versus SHR + saline group

**Table 4:** Moving speed in the open field test (mean ± SEM, cm/s, n = 10)

Group	Week 0	Week 1	Week 2	Week 3	Week 4
WKY + saline	7.09 ± 0.68	6.71 ± 1.25	6.00 ± 1.16	4.66 ± 1.03	4.01 ± 1.28
SHR + saline	20.46 ± 1.45^##^	17.37 ± 0.91^##^	18.51 ± 1.03^##^	17.65 ± 1.34^##^	18.23 ± 1.54^##^
SHR + MPH(1.5 mg/kg)	18.73 ± 1.07	11.56 ± 1.10^**^	8.81 ± 1.29^**^	6.80 ± 1.45^**^	4.10 ± 1.42^**^
SHR + baicalin(50 mg/kg)	18.36 ± 1.00	16.75 ± 1.67	15.93 ± 1.50	11.08 ± 0.47^*^	9.98 ± 1.29^**^
SHR + baicalin(100 mg/kg)	19.75 ± 1.61	16.54 ± 1.06	10.16 ± 0.89^**^	8.76 ± 1.17^**^	5.95 ± 0.72^**^
SHR + baicalin(150 mg/kg)	18.15 ± 1.14	13.41 ± 0.98^*^	9.12 ± 1.02^**^	6.84 ± 1.92^**^	4.64 ± 1.14^**^

Note: Comparison of the moving speed in open field test in all groups; values are expressed as the mean ± S.E.M. ^#^*p* < 0.05 and ^##^*p* < 0.01 versus WKY + saline group; ^*^p < 0.05 and ^**^p < 0.01 versus SHR + saline group

After two weeks of treatment, the moving distance and moving speed were significantly decreased in the MPH + saline and all baicalin-treated groups (moving distance, MPH, *p* < 0.01; 150 mg/kg, *p* < 0.01; 100 mg/kg, *p* < 0.05; LSD), (moving speed, MPH, *p* < 0.01; 150 mg/kg, *p* < 0.01; 100 mg/kg, *p* < 0.01; LSD) compared with the SHR + saline group.

After three weeks of treatment, the MPH + saline and all baicalin treated groups showed decreased moving distance (MPH, *p* < 0.01; 150 mg/kg, *p* < 0.01; 100 mg/kg, *p* < 0.01, 50 mg/kg, *p* < 0.05; LSD); the MPH + saline and baicalin groups at all dose showed decreased in moving speed (MPH, *p* < 0.01; 150 mg/kg, *p* < 0.01; 100 mg/kg, *p* < 0.01; 50 mg/kg, *p* < 0.05; LSD) compared to the SHR + saline group.

**Fig. 1:**
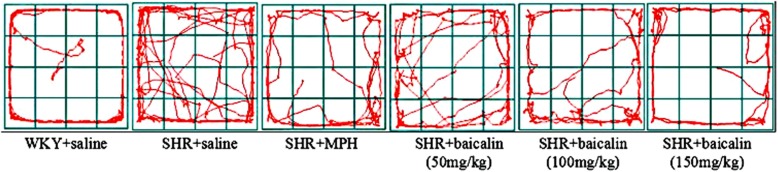
Typical trajectory in the open field test (means±SEM, n = 10). Note: Typical moving trajectory in the open field test

After treatment for four weeks, the MPH + saline and all baicalin treated groups showed a significant decrease in moving distance and moving speed (MPH, *p* < 0.01; 150 mg/kg, *p* < 0.01; 100 mg/kg, *p* < 0.01, 50 mg/kg, *p* < 0.01; LSD) compared to the SHR + saline group; typical trajectories are shown in Fig. 1.

#### Baicalin treatment-induced improvements in spatial learning and memory

**Fig. 2:**
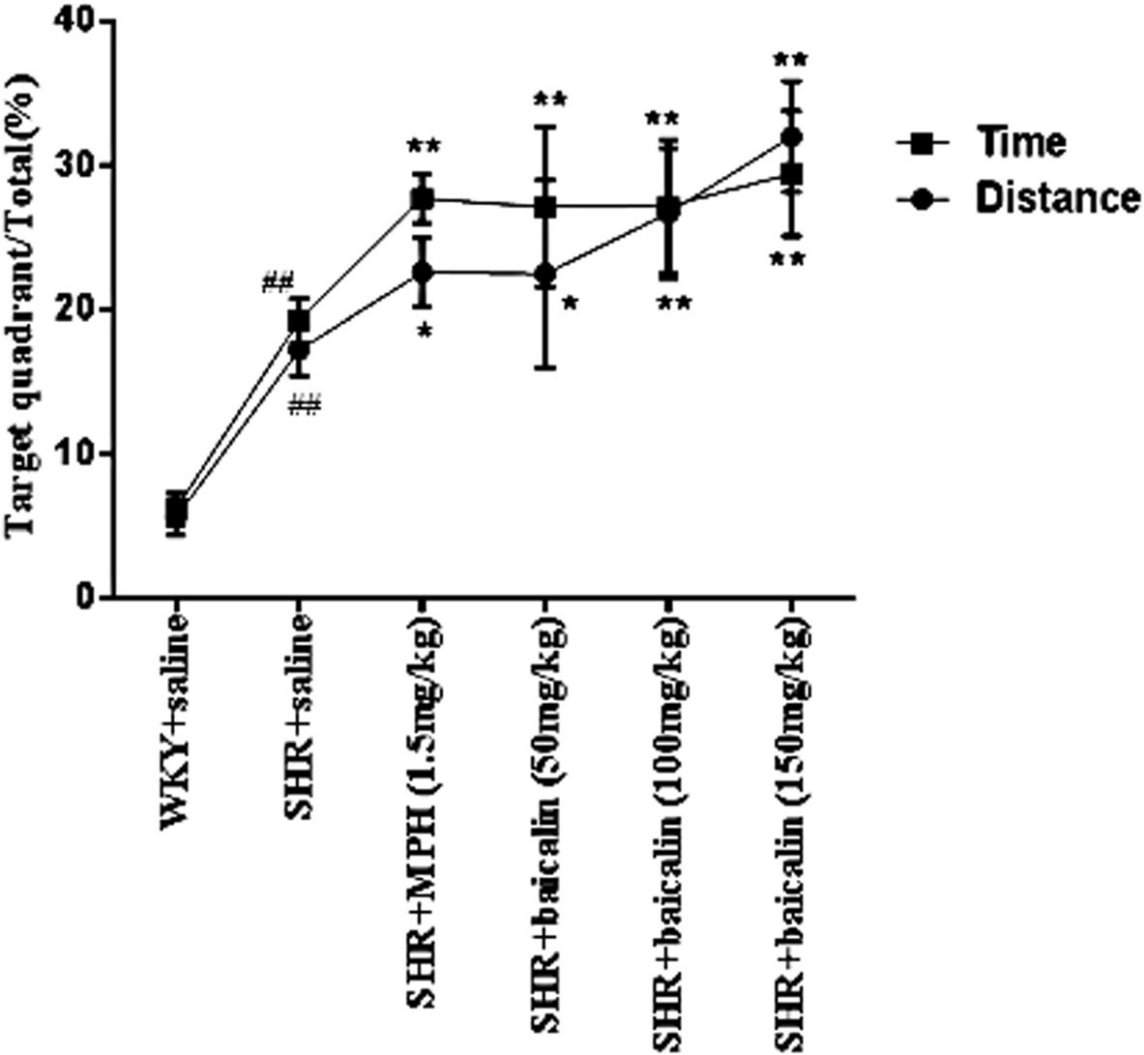
Target quadrant (southeast quadrant where the platform was located) swimming time and distance to the total time and distance (mean ± SEM, %, n = 10). Note: ^#^*p* < 0.05 and ^##^*p* < 0.01 versus WKY + saline group; ^*^p < 0.05 and ^**^p < 0.01 versus SHR + saline group

**Fig. 3:**
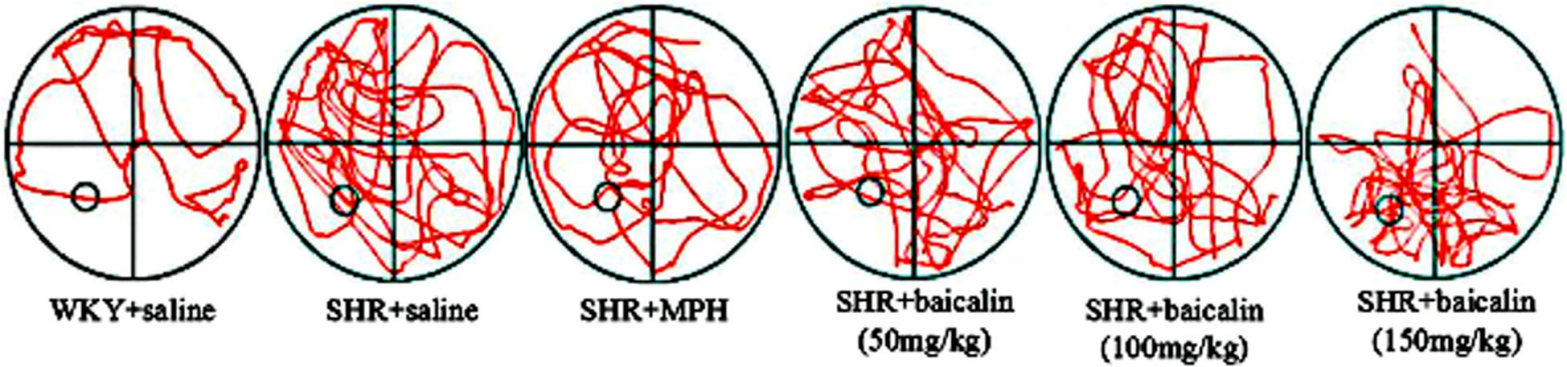
Typical trajectory in the Morries Water Maze test (mean ± SEM, n = 10). Note: Typical swimming trajectory in the Morries Water Maze test

All latencies for the different groups are shown in Table 5. Repeated measures ANOVA showed a between-subject effect in escape latency (F_(5,55)_ = 79.586, *p* < 0.001). Later, LSD test were used. During acquisition training, SHR + saline rats required more time to find the platform than the WKY + saline did during the entire test (Tables 5 and 6). During all of the training days, all of the drug-treated SHRs required significantly less time (escape latency; *p* < 0.01) to find the platform compared with saline-treated SHRs. After the second and subsequent training days, the SHRs treated with MPH (*p* < 0.01 for distance) and 150 mg/kg and 100 mg/kg of baicalin (*p* < 0.05 for distance and both doses) showed shorter swimming distances compared with saline-treated SHRs. SHRs treated with 50 mg/kg of baicalin showed significantly shorter swimming distances (*p* < 0.01) compared with saline-treated SHRs after the fourth and fifth days of training. As shown in Fig. 2, saline-treated SHRs had significantly higher ratios of target quadrant swimming time and distance to the total swimming time and distance (*p* < 0.01) compared with WKY rats. SHRs that were treated with MPH and all baicalin doses also showed significantly higher ratios (*p* < 0.01 or *p* < 0.05) compared with saline-treated SHRs. The typical trajectories are shown in Fig. 3.

**Table 5:** Escape latency in the Morries Water Maze test (mean ± SEM, s, *n* = 10)

Group	Day1	Day2	Day3	Day4	Day5
WKY + saline	56.14 ± 3.51	48.71 ± 4.30	45.01 ± 3.69	39.94 ± 2.62	40.78 ± 1.52
SHR + saline	68.22 ± 3.13^##^	61.96 ± 3.89^##^	54.51 ± 4.89^##^	54.76 ± 4.63^##^	50.82 ± 5.49^#^
SHR + MPH(1.5 mg/kg)	53.96 ± 1.92^**^	36.18 ± 2.37^**^	32.18 ± 2.24^**^	25.28 ± 2.18^**^	21.82 ± 2.33^**^
SHR + baicalin(50 mg/kg)	50.83 ± 4.40^**^	46.65 ± 2.95^**^	40.88 ± 2.67^**^	41.35 ± 1.73^**^	38.72 ± 2.03^**^
SHR + baicalin(100 mg/kg)	49.82 ± 2.98^**^	37.17 ± 2.38^**^	33.34 ± 2.24^**^	32.87 ± 1.58^**^	32.58 ± 1.78^**^
SHR + baicalin(150 mg/kg)	45.37 ± 3.41^**^	40.49 ± 2.36^**^	33.02 ± 3.19^**^	27.81 ± 2.72^**^	24.87 ± 1.85^**^

Note: Comparison of the escape latency in Morries water maze in all groups; values are expressed as the mean ± S.E.M. ^#^*p* < 0.05 and ^##^*p* < 0.01 versus WKY + saline group; ^*^p < 0.05 and ^**^p < 0.01 versus SHR + saline group

**Table 6:** Swimming distance in the Morries Water Maze test (mean ± SEM, m, n = 10)

Group	Day1	Day2	Day3	Day4	Day5
WKY + saline	6.00 ± 1.42	4.25 ± 0.85	5.14 ± 1.19	5.98 ± 1.33	5.76 ± 0.68
SHR + saline	13.54 ± 0.83^##^	11.00 ± 1.06^##^	9.11 ± 1.20^##^	9.15 ± 1.05^#^	9.45 ± 1.12^#^
SHR + MPH(1.5 mg/kg)	12.96 ± 1.09	8.15 ± 1.12^*^	4.46 ± 0.90^**^	3.35 ± 1.14^**^	3.25 ± 0.91^**^
SHR + baicalin(50 mg/kg)	12.53 ± 0.88	10.18 ± 0.32	7.76 ± 1.05	4.49 ± 1.14^**^	5.30 ± 1.39^**^
SHR + baicalin(100 mg/kg)	11.18 ± 1.04	8.09 ± 1.56^*^	5.57 ± 1.26^**^	4.15 ± 1.16^**^	4.67 ± 1.40^**^
SHR + baicalin(150 mg/kg)	12.90 ± 0.42	7.22 ± 1.18^**^	4.42 ± 1.21^**^	3.25 ± 1.27^**^	3.15 ± 1.01^**^

Note: Comparison of the swimming distance in Morries water maze in all groups, values are expressed as the mean ± S.E.M. ^#^*p* < 0.05 and ^##^*p* < 0.01 versus WKY + saline group; ^*^p < 0.05 and ^**^p < 0.01 versus SHR + saline group

### Typical synaptosomes

**Fig. 4:**
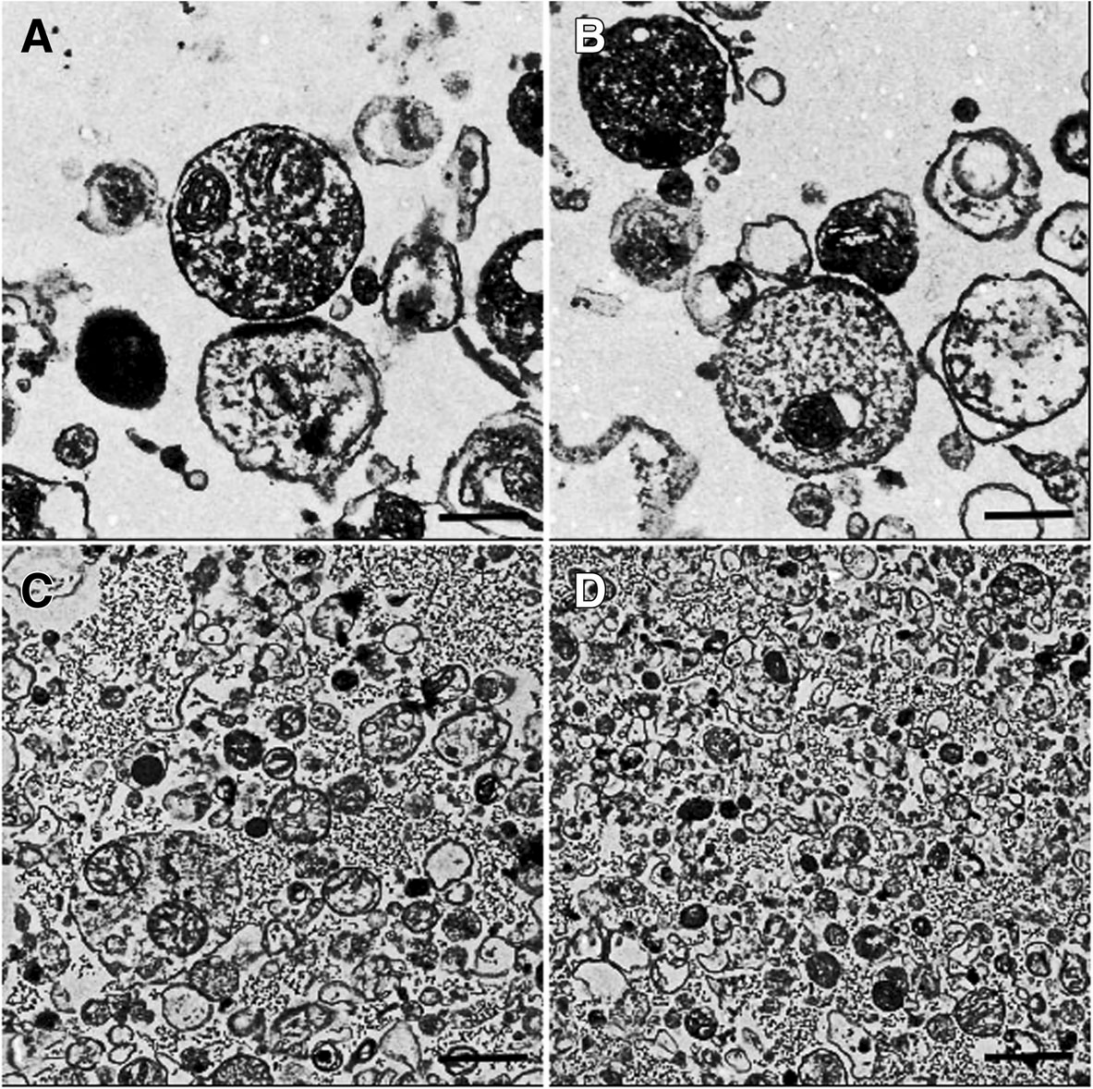
Typical synaptosomes. Note: Typical synaptosomes examined by electron microscopy (EM). Synaptosomes were prepared with a Percoll gradient procedure. EM showed the presence of intact synaptic vesicles and the preservation of presynaptic and postsynaptic elements and a higher mitochondrial content and contained a few extrasynaptic mitochondria. Scale bar is 0.5 μm in (**a** and **b)** and 1 μm in (**c** and **d**)

As described previously [26, 27], electron microscopic examinations showed that the synaptosomes exhibited numerous synaptic vesicles and a higher mitochondrial content. In addition, they contained extrasynaptic mitochondria, and the presynaptic and postsynaptic elements were preserved, as shown in Fig. 4 (A-D).

#### Effects of baicalin on the synaptosomal protein levels of TH, SNAP25, VMAT2, and syntaxin 1a

**Fig. 5:**
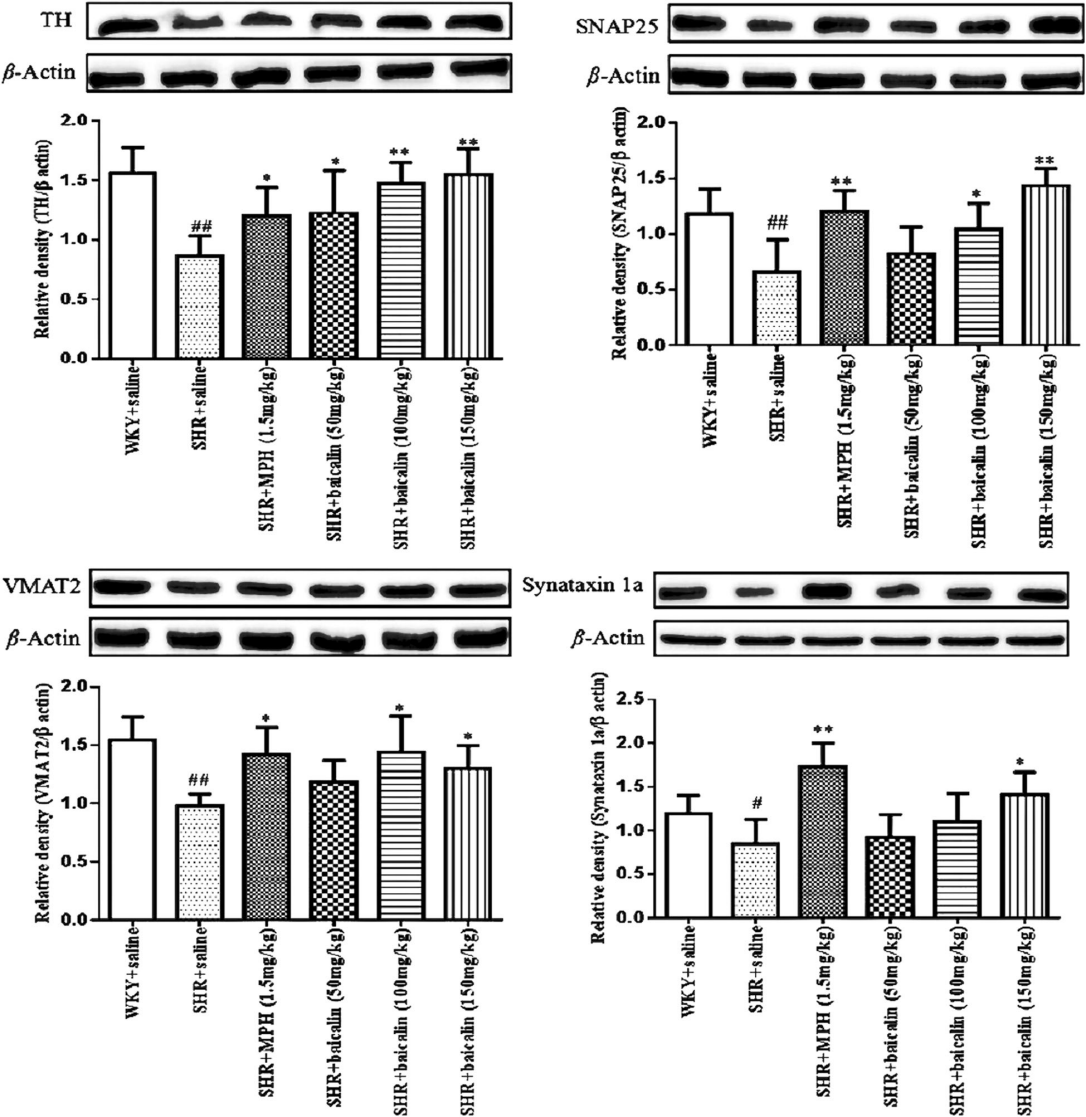
The protein levels of TH, SNAP25, VMAT2 and synataxin 1a in synaptosomes. Note: Effects of baicalin on the protein expression of TH, SNAP25, VMAT2 and Synataxin 1a in synaptosomes. Data are expressed as the mean ± SEM, n = 10. ^#^*p* < 0.05 and ^##^*p* < 0.01 versus WKY + saline group; ^*^p < 0.05 and ^**^p < 0.01 versus SHR + saline group

All datas for the different groups were tested by one-way ANOVA and LSD. As shown in Fig. 5, the protein levels of TH, SNAP25, VMAT2 and synataxin 1a were significantly decreased ((*F*_(5,55)_ = 7.099, *p* < 0.001) for TH, SNAP25, VMAT2 and (*F*_(5,55)_ = 3.238, *p* = 0.015) for synataxin 1a) in the saline-treated SHRs compared with the WKY rats. MPH-treated and baicalin (150 mg/kg)-treated SHRs had significantly increased protein levels of TH, SNAP25, VMAT2, and syntaxin 1a (*p* < 0.01 for TH, SNAP25, synataxin 1a and *p* < 0.05 for VMAT2; LSD) compared with the levels in saline-treated SHRs. The protein levels of TH, SNAP25, and VMAT2 were significantly increased (*p* < 0.01 for TH and *p* < 0.05 for SNAP25, VMAT2; LSD) in baicalin (100 mg/kg)-treated SHRs compared with saline-treated SHRs. Compared with saline-treated SHRs, only the levels of TH were significantly increased (*p* < 0.05, LSD) in baicalin (50 mg/kg)-treated SHRs.

#### Effects of baicalin on the synaptosomal mRNA levels of TH, SNAP25, VMAT2 and syntaxin 1a

**Fig. 6:**
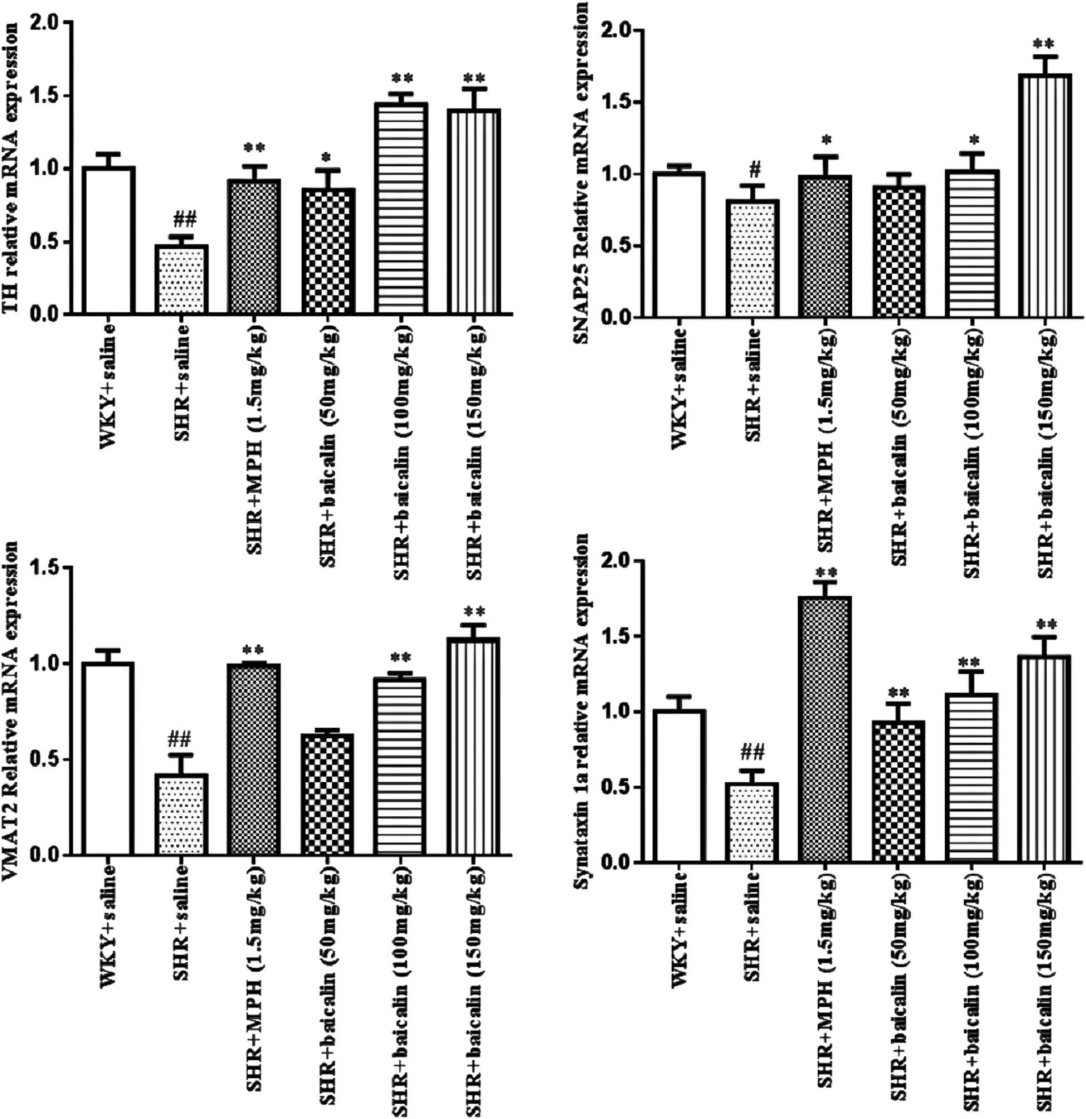
mRNA levels of TH, SNAP25, VMAT2, and syntaxin 1a in synaptosomms. Note: Effects of baicalin on the mRNA expression of TH, SNAP25, VMAT2 and synataxin 1a in synaptosomes. Data are expressed as the mean ± SEM, *n* = 10. ^#^*p* < 0.05 and ^##^*p* < 0.01 versus WKY + saline group; ^*^p < 0.05 and ^**^p < 0.01 versus SHR + saline group

All datas for the different groups were tested by one-way ANOVA and LSD. As shown in Fig. 6, the mRNA levels of TH (*F*_(5,55)_ = 13.189, *p* < 0.001), SNAP25 (*F*_(5,55)_ = 31.046, *p* < 0.001), VMAT2 (*F*_(5,42)_ = 3.592, *p* = 0.012), and syntaxin 1a (*F*_(5,55)_ = 24.921, *p* < 0.001) were significantly decreased (*p* < 0.01 for TH, VMAT2, synataxin 1a and *p* < 0.05 for SNAP25; LSD) in saline-treated SHRs compared with WKY rats. SHRs that were treated with MPH and baicalin (150 mg/kg and 100 mg/kg) showed markedly upregulated levels of TH, SNAP25, VMAT2, and syntaxin 1a mRNA (*p* < 0.01 for TH, VMAT2, synataxin 1a and *p* < 0.05 for SNAP2; LSD) compared with saline-treated SHRs. SHRs treated with 50 mg/kg of baicalin exhibited significantly increased mRNA levels of TH and syntaxin 1a (*p* < 0.01 for synataxin 1a and *p* < 0.05 for TH; LSD) compared with SHRs treated with saline.

#### Effects of baicalin on DA levels in the PFC and striatum

**Fig. 7:**
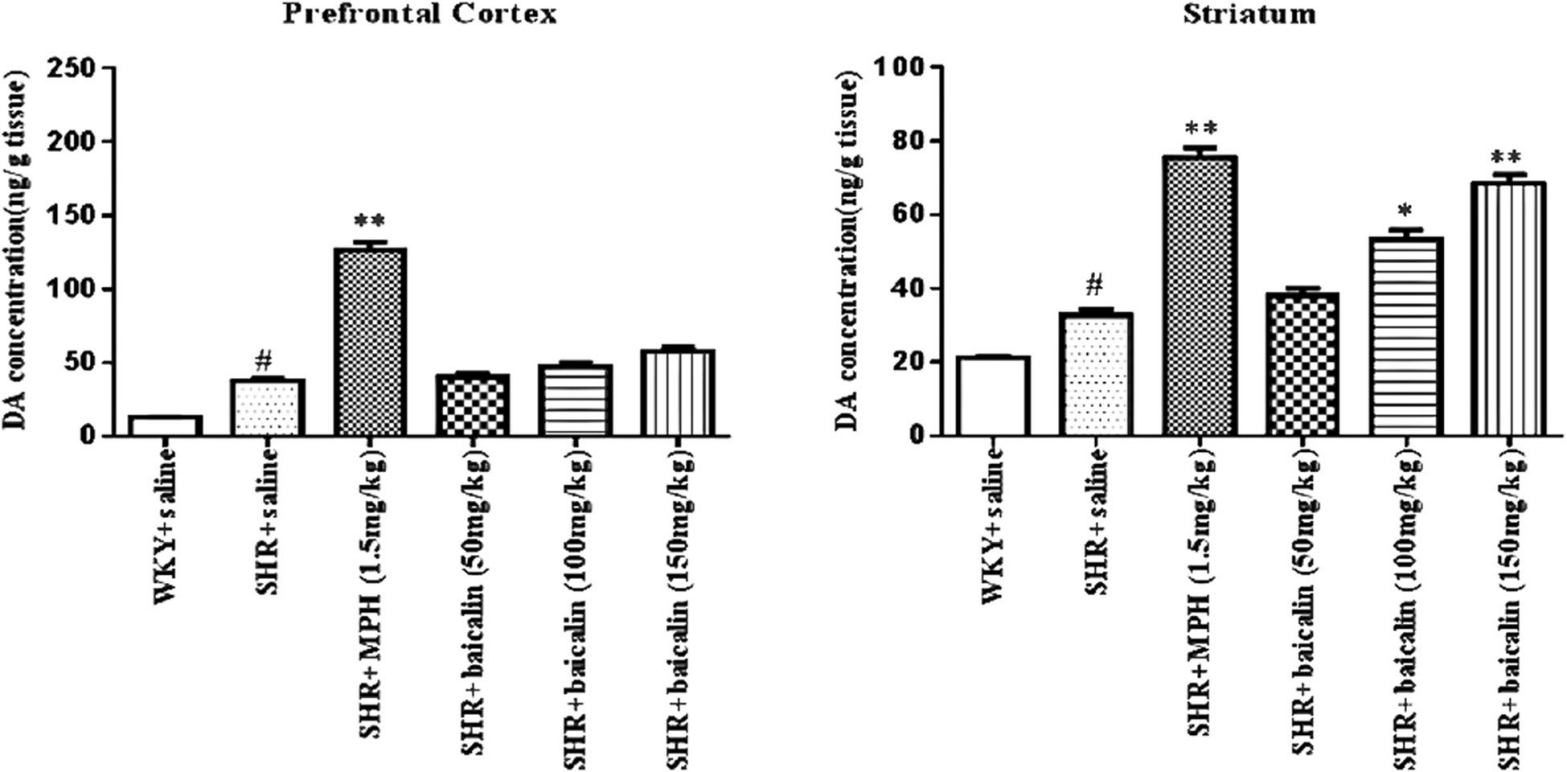
DA levels in the PFC and Striatum. Note: Effects of baicalin on the DA levels in the PFC and striatum. A and B represent DA concentration in PFC and striatum. Data are expressed as the mean ± SEM, n = 10. ^#^*p* < 0.05 and ^##^*p* < 0.01 versus WKY + saline group; ^*^p < 0.05 and ^**^p < 0.01 versus SHR + saline group

All datas for the different groups were tested by one-way ANOVA and LSD. As summarized in Fig. 7, DA levels were significantly increased in the PFC and striatum (*p* < 0.05 for both) of saline-treated SHRs compared with WKY rats. Compared with the saline-treated SHRs, the DA levels in the PFC and striatum of the MPH-treated SHRs were significantly increased (*p* < 0.01 for both). The SHRs that were treated with 150 mg/kg and 100 mg/kg of baicalin had increased levels of DA in the striatum (*p* < 0.01 and 0.05, respectively).

## Discussion

In a previous study, we hypothesized that baicalin would have therapeutic effects in patients with ADHD [23]. To investigate this hypothesis, we investigated if baicalin decreased ADHD symptoms and its underlying mechanisms in 4-week-old SHRs in this study, as 4-week-old rats are developmentally equivalent to the beginning of human childhood [28]. SHRs are one of the most frequently used animal models of ADHD worldwide because they display symptoms similar to the core symptoms of ADHD, such as hyperactivity, impulsivity, and poorly sustained attention compared with WKY control rats [29, 30]. For drug safety considerations during the course of the study, we measured changes in body weight and food intake in rats to evaluate the effect of baicalin on the growth and development of rats. The results showed that baicalin did not affect the normal eating and weight gain of rats, demonstrating that baicalin was safe for gavage.

The core clinical symptoms of ADHD are hyperactivity, impulsivity, and inattention. Whether baicalin can control the core symptoms of ADHD is the basic condition for verifying our hypothesis. In previous studies, moving distance and moving speed were often recorded by the open-field test method to evaluate the hyperactivity behavior in ADHD [31]. The MWM can be used to test spatial learning in rodents, and it has been widely used to test the attention and learning abilities of SHR rats [32]. We evaluated the effects of baicalin on the regulation of behavior in SHRs. In accordance with previous studies, saline-treated SHRs showed increased moving distance and moving speed, which represents typical hyperactivity symptoms, compared with WKY rats, as shown in Tables 3 and 4. After treating the SHRs with baicalin and MPH, these rats showed significant reductions in locomotion. In tests of the spatial learning abilities of the SHR rats, the SHRs treated with MPH and baicalin (especially 150 mg/kg and 100 mg/kg) were faster to find the platform and showed increased ratios in the target quadrant than the saline-treated SHRs (Tables 5 and 6). The typical trajectories showed that the SHRs treated with MPH and baicalin (especially 150 mg/kg and 100 mg/kg) presented significant thigmotaxis movement loci, which is a tendency to move closer to the wall, compared to the WKY rats, while the saline-treated SHRs displayed a chaotic movement trajectory (Figs. 1 and 3) [33]. The behavioral tests showed that treatment with MPH and baicalin (especially 150 mg/kg and 100 mg/kg) significantly reduced the locomotion and increased the spatial learning abilities of SHRs compared with saline-treated SHRs, thus showing effects on the regulation of SHRs behavior.

During the MWM test, WKY rats often floated on the water at the moment they were released into the water and during the experiment. Previous studies have also reported this phenomenon [34]. This phenomenon contrasts with rats’ typical fear of water. Thus, some scholars have suggested that WKY rats have abnormal behaviors and exhibit different degrees of depressive symptoms [35]. One possible reason is the presence of sensory abnormalities in WKY rats. In the study of hypertension, WKY rats are not sensitive to sound stimuli compared with SHRs [36]. Further research is needed to be done to prove whether WKY rats prefer swimming more than other rats or if there is a tactile abnormality in WKY rats.

According to the DA deficit theory, ADHD symptoms might be related to disturbed DA neurotransmission; therefore, we examined the DA system. DA is a monoamine neurotransmitter in the brain; the entire process of dopamine synthesis, release, and removal requires the synaptic vesicles in the synaptosomes. Therefore, the synaptosome is a key structure in the study of the DA system [37, 38]. In synaptosomes, abnormalities in the function of dopamine transporters and D1/D2 receptors in the brain have been reported [39, 40]. Recent research found that DA synthesis, vesicular localization and release are dynamically regulated by several factors, including tyrosine hydroxylase (TH), vesicular monoamine transporter 2 (VMAT2), synaptosomal-associated protein 25 (SNAP25), and syntaxin 1a [41, 42]. TH is the rate-limiting enzyme in the conversion of L-tyrosine to 3,4-dihydroxy-L-phenylalanine (L-DOPA), which is ultimately converted to DA. DA is then transported from the cytoplasm into synaptic vesicles by VMAT2, which is expressed in presynaptic terminals. Syntaxin 1a and SNAP-25 mediate the anchoring and fusion of vesicles and presynaptic membranes and the release of DA into the synaptic cleft. The released DA can bind to DA receptors in the presynaptic and postsynaptic membranes. DA is then taken back up into dopaminergic terminals by the dopamine transporter in the presynaptic membrane and then degraded by monoamine oxidase. An alteration in any of these factors may ultimately disturb the metabolism, transport, and utilization of DA and lead to DA deficits in the brain. As shown in this study, saline-treated SHRs showed significantly decreased protein and mRNA levels of TH, SNAP25, VMAT2, and syntaxin 1a compared with WKY rats. After treatment with MPH and baicalin (especially the 150 mg/kg and 100 mg/kg doses), SHRs showed significantly increased protein and mRNA levels of TH, SNAP25, VMAT2, and syntaxin 1a compared with saline-treated SHRs (Figs. 5 and 6). These results indicated that the pharmacological effects of baicalin were largely associated with DA synthesis, vesicular localization, and release and suggested that baicalin might significantly influence DA levels in the brain. We, therefore, measured the DA levels in the PFC and striatum, which are two key brain regions closely related to the dopamine system and the onset of ADHD [43, 44]. As shown in Fig. 7, MPH can simultaneously increase DA content in the prefrontal and striatum regions, while baicalin at the doses of 150 mg/kg and 100 mg/kg only significantly increased DA levels in the striatum and had little influence on the PFC. The biochemical action of MPH is to block the dopamine transporter (DAT) and norepinephrine transporter (NET), which results in an elevated concentration of dopamine (DA) and norepinephrine (NA) at synapses to control the symptoms of ADHD [45, 46]. The PFC is a key brain region mediating cognitive and executive functions, such as working memory, sustained attention, inhibitory response control, and cognitive flexibility [43, 47]. ADHD patients have shown delayed maturation in the PFC [48], dysfunction of the frontostriatal circuitry [49], and hypoactivation in the frontal cortex [50, 51]. Thus, researchers found that the PFC is identified as the primary target of MPH [52]. Commonly, MPH acts on attention and cognition through an increase in D in the striatum; the conventional dose of MPH has an almost marginally significant increase in the PFC [53]. However, some researches has found that a therapeutic dose of MPH acutely improves cognitive functions by modifying the function of SNAP25 and glutamate receptors in the PFC, while an overdose of MPH inhibited this function and induced psychosis; the PFC is the primary target of a therapeutic dose of MPH worked on [54].

In this study, MPH increased the DA content in both regions, meanwhile, with activation of SNAP25/VMAT2. We speculate that administration of MPH for 4 consecutive weeks may affect the PFC since MPH has long-lasting metaplastic effects in the PFC [55]. Though no glutamate receptor was detected in this study, the activation status of SNAP25/VMAT2 is clearly and the state of glutamate receptors cannot be ruled out. A previous study from Lujun Zhang [56] proved that after treatment with baicalin, baicalin passed through the blood–brain barrier and distributed within the brain tissue, specifically in the hippocampus, striatum, cortex, and thalamus, although the exact mechanism was not reported [57]. Research has found that baicalin can regulate the GABA receptor and inflammatory pathway in the PFC, suggesting that the PFC may also be the target region for baicalin [58, 59]. However, recent researches on the pharmacokinetics of baicalin showed the baicalin content in brain’s target was higher in the striatum and cerebellum, the striatum is the preferential distribution location of baicalin [60] and this maybe the reason why baicalin showed little influence on the DA concentration in the prefrontal cortex region. Thus, we must examine whether the striatum region is the “target region” of baicalin. This topic will be a new direction in further research. We will use more animal experiments to prove our theory in the further. We will use more animal experiments to prove it in the future. Besides, a recent research by our group showed that baicalin has the potent to depress the expression of DAT mRNA as well as protein in SHR rat, to block the reuptaking DA process performed by presynaptic neuron, and then to increase the DA concentration to improve the conduction of DA in the synaptic gap. [Rongyi Zhou, et al. (submitted) Effect of baicalin on the expression of dopamine transporter in SHR rats in striatum synaptosome]. All of the above results indicated that baicalin affected the core symptoms of ADHD by modifying the regulation of DA synthesis, vesicular localization, and release and increased DA levels in the striatum.

This study provides evidence that baicalin regulates DA synthesis and release, increases DA levels in the striatum, and controls the core symptoms of ADHD [61–63]. However, the results of this study raise many questions that need to be resolved. First, further research is needed on the optimal dose of baicalin for controlling the core symptoms of ADHD and regulating DA synthesis, vesicular localization, and release. Second, during the MWM test, the WKY rats usually floated on the water, which is contrary to the physiological characteristics of rats, which are afraid of water [64, 65]. Therefore, this phenomenon needs to be examined further in future research. Third, we need to further investigate why baicalin had little effect on the DA levels in the PFC, while it significantly increased the DA levels in the striatum. Is the target of baicalin action located in the striatum? Other synaptic-associated proteins might be useful in these future investigations. Synaptophysin, which is a synaptic vesicle glycoprotein expressed in neuroendocrine cells and most neurons in the CNS, is a hallmark of synaptic vesicle maturation that is considered an indirect marker of synaptogenesis in the developing brain [66]. Similarly, postsynaptic density protein 95, which is involved in the maturation of excitatory synapses and brain-derived neurotrophic factor, which is related to synaptic plasticity [67], are associated with synaptosomal development and the onset of ADHD [68, 69]. Thus, the roles of these proteins should be examined in future studies.

We know that the current research content and mechanism explanation is superficial. However, we aim to publish the research so that it can be seen by more people for continued research that may be helpful for ADHD children. A better understanding of ADHD can help with treatment of children with ADHD and improve quality of life for these children and their families.

## Abbreviations

AD: Alzheimer’s disease
ADHD: Attention deficit hyperactivity disorder
BBB: Blood-brain barrier
CNS: Central nervous system
DA: Dopamine
DAT: Dopamine transporter
HPLC: High performance liquid chromatography
L-DOPA: 3,4-dihydroxy-L-phenylalanine
MPH: Methylphenidate
MWM: Morris water maze
PCR: Polymerase chain reaction
PFC: Prefrontal cortex
SEM: Standard error of the mean
SHR: Spontaneously hypertensive rat
SNAP25: Synaptosomal-associated protein 25
TH: Tyrosine hydroxylase
VMAT2: Vesicular monoamine transporter 2
WKY: Wistar Kyoto
